# Description of the fasted serum metabolomic signature of lean and obese cats at maintenance and of obese cats under energy restriction

**DOI:** 10.1371/journal.pone.0299375

**Published:** 2024-03-15

**Authors:** Caitlin E. Grant, Hannah Godfrey, Moran Tal, Marica Bakovic, Anna K. Shoveller, Shauna L. Blois, Myriam Hesta, Adronie Verbrugghe

**Affiliations:** 1 Department of Clinical Studies, Ontario Veterinary College, University of Guelph, Guelph, Ontario, Canada; 2 Department of Biomedical Sciences, Ontario Veterinary College, University of Guelph, Guelph, Ontario, Canada; 3 Department of Human Health and Nutritional Sciences, College of Biological Sciences, University of Guelph, Guelph, Ontario, Canada; 4 Department of Animal Biosciences, Ontario Agricultural College, University of Guelph, Guelph, Ontario, Canada; 5 Department of Morphology, Imaging, Orthopedics, Rehabilitation and Nutrition, Faculty of Veterinary Medicine, Ghent University, Merelbeke, Belgium; Inner Mongolia University, CHINA

## Abstract

This study aimed to investigate the serum metabolomic profile of obese and lean cats as well as obese cats before and after energy restriction for weight loss. Thirty cats, 16 obese (body condition score 8 to 9/9) and 14 lean (body condition score 4 to 5/9), were fed a veterinary weight loss food during a 4-week period of weight maintenance (L-MAINT and O-MAINT). The 16 obese cats were then energy restricted by a 60% energy intake reduction with the same food for a 10-week period (O-RESTRICT). Fasted serum metabolites were measured using nuclear magnetic resonance and direct infusion mass spectrometry after the maintenance period for L-MAINT and O-MAINT cats and after the energy restriction period for O-RESTRICT and compared between groups using a two-sided t-test. Obese cats lost 672 g ± 303 g over the 10-week restriction period, representing a weight loss rate of 0.94 ± 0.28% per week. Glycine, l-alanine, l-histidine, l-glutamine, 2-hydroxybutyrate, isobutryric acid, citric acid, creatine, and methanol were greater in O-RESTRICT compared to O-MAINT. There was a greater concentration of long-chain acylcarnitines in O-RESTRICT compared to both O-MAINT and L-MAINT, and greater total amino acids compared to O-MAINT. Glycerol and 3-hydroxybutyric acid were greater in O-MAINT compared to L-MAINT, as were several lysophosphatidylcholines. Thus, energy restriction resulted in increased dispensable amino acids in feline serum which could indicate alterations in amino acid partitioning. An increase in lipolysis was not evident, though greater circulating acylcarnitines were observed, suggesting that fatty acid oxidation rates may have been greater under calorie restriction. More research is needed to elucidate energy metabolism and substrate utilization, specifically fatty acid oxidation and methyl status, during energy restriction in strict carnivorous cats to optimize weight loss.

## Introduction

Obesity is a major health concern for domestic cats [1, 2], but the underlying metabolic and biochemical processes are not well understood. These mechanisms can be further explored through the investigation of the *metabolome*, the measurement of small molecule metabolites, their precursors, derivatives, and degradation products [3, 4]. The application of metabolomics in the field of nutrition is increasing and relates to two main research categories including dietary intervention studies and identification of biomarkers for disease [5, 6].

In cats, metabolomic technologies are highly underutilized compared to other species for dietary interventions [7–13] and disease states [14–16], with only one study to the authors’ knowledge assessing the serum metabolome in overweight and obese cats [11]. Said study did, however, not have a lean comparison. In both humans, rodents, dogs, and cats differences have been observed between obese and lean individuals [17–25]. In humans, one study in particular used a non-targeted metabolomics approach and revealed that nearly one third of metabolites analyzed were associated with body mass index [17]. Similarly, Forster et al., observed 185 plasma metabolites that were different between lean and overweight or obese dogs [18]. Of note, branched-chain amino acids (BCAA), glutamine, methionine, and phenylalanine, the lysophosphatidylcholine (LPC) C18:1 and several acylcarnitines have been identified as potential biomarkers of obesity in humans, rodents, and dogs [18–24]. An increase in BCAA is suggested to reflect an increased BCAA release from protein breakdown; however, with markers for impaired fatty acid oxidation, an increase in BCAA could suggest inhibition of the branched chain keto acid dehydrogenase enzyme, which would cause increased BCAA in the muscle due to reduced catabolism, and release into circulation [26]. Additionally, increases in medium and long chain acylcarnitines have previously been observed in obese individuals which was suggested to indicate impaired fatty acid oxidation [27]. Overall, it is unclear whether these findings can be extrapolated to cats, an obligate carnivore, due to their metabolic differences. In a comparison between dogs and cats, it was observed that 76% of the metabolites analyzed trended differently between the two species [10]; specifically, alterations were noted in circulating lipids and plasma BCAA concentrations were reported to be higher in cats than in dogs, likely due to higher gluconeogenic activity in the cat.

The metabolome was also investigated in relation to calorie restriction in both obese [5] and healthy weight [4] individuals. Obese humans consuming a low-calorie diet for 12 weeks had greater medium- and long-chain acylcarnitine and free fatty acid concentrations compared to an obese control group consuming a maintenance diet [5]. Lipolysis of visceral fat generates free fatty acids, which drive the rate of fatty acid oxidation, and thus increase the influx of medium- and long-chain acylcarnitines. In agreement, healthy weight individuals subject to an acute dietary energy restriction were again shown to have greater serum levels of glycerol, decreased monoacylglycerols, an increase of all long and most medium-chain fatty acids and an increase in long-chain acylcarnitines together suggesting greater amounts of lipolysis [4]. Interestingly, overweight and obese cats undergoing energy restriction for weight loss at a 1.5% body weight (BW) reduction per week using a moderate protein, high fibre diet for 16 weeks showed decreases in long-chain fatty acids, though medium-chain fatty acids increased [28]. Additional inconsistencies with results from calorie restriction in humans include an increase in monoacylglycerols and greater concentrations for markers of acylcarnitine metabolism [28]. Moreover, the ketone bodies, acetoacetate and 3-hydroxybutyrate (BHB), were greater with energy restriction than maintenance energy provision in humans, indicating greater β-oxidation [4] which is consistent with previous work in overweight and obese cats undergoing energy restriction [28]. As energy restriction resulted in a greater reliance on fatty acids for energy production in humans, carbohydrate utilization decreased, as noted by a decrease in serum glucose and pyruvate [4]. In cats, metabolites for carbohydrate metabolism were reduced, though this was likely due to the overall reduced food intake [28]. Finally, energy restriction resulted in N-acetyl derivate of the gluconeogenic AA, glycine, serine and alanine and greater BCAA metabolism in humans [4]. The BCAAs, leucine, isoleucine and valine, as well as BCAA catabolites were increased with energy restriction [4], though it is unclear whether this is a direct effect of energy restriction, or a secondary effect of energy restriction altering partitioning of macronutrients, resulting in lean tissue loss to support energy metabolism. Alternatively, after 16 weeks of calorie restriction in overweight and obese cats, there were no changes in serum metabolites of BCAA metabolite concentrations [28]. This difference between cats and humans could be due to a cats’ higher requirement for protein and balance between muscle mass loss and muscle synthesis.

To date and to the authors’ knowledge, Pallotto et al., is the only available metabolomics study investigating the metabolic profile of overweight and obese cats and the effects of weight loss via energy restriction [28]. However, this study is small in sample size (n = 8) with no lean control group and all cats were neutered males. Further, cats had a mean body condition score (BCS) of 7.6 out of 9, indicating that cats were a mix of overweight and obese, which could have reduced the ability to observe changes in the metabolomic signature. Research is needed to support the use of metabolomics to detect obesity-induced changes in metabolism as well as to help identify biomarkers that may be related to obesity and its co-morbidities. Furthermore, cats are strict carnivores and preliminary research has identified that there may be differences in the circulating metabolome associated with obesity and energy restriction in cats from what is observed in omnivorous mammals, such as humans, rodents and dogs. This study aimed to first identify and compare the metabolomic signature of lean and obese cats fed to maintenance and of obese cats after a period of energy restriction for weight loss using a therapeutic weight loss diet. Second, this study aimed to use metabolomic technologies to generate hypotheses regarding obesity and energy restriction in cats due to the lack of available research. That said, the authors’ hypothesized that the metabolomic signature of obese cats would show signs of dyslipidemia and altered fatty acid oxidation compared to lean cats, though BCAAs would be similar between groups. Further, it is hypothesized that energy restriction resulting in weight loss in the obese group would show shifts in fatty acids and amino acids and their metabolites such as increased fatty acid oxidation and gluconeogenic activity.

## Materials and methods

The study protocol adhered to the University of Guelph Animal Use Protocol (AUP), was approved by the University of Guelph Animal Care Committee (#AUP 2496) and were in accordance with national and institutional guidelines for the care and use of animals in research. Owners of the cats gave written informed consent for participation in the study.

### Study design

The study design has been described in detail in a previous publication [29]. Briefly, 30 client owned cats were enrolled in the study and fed a dry commercial veterinary therapeutic cat food intended for maintenance and weight loss between May 2015 and December 2016. Cats were divided into two groups based on BCS. Cats with a BCS of 4 to 5/9 were assigned to the lean group (n = 14; 10 males, 4 females; initial mean BW ±SEM = 4.37±0.83) and cats with a BCS of 8 to 9/9 were assigned to the obese group (n = 16; 10 males, 6 females; initial mean BW ±SEM = 7.05±1.43) according to the 2011 WSAVA guidelines [30]. Following a 1-week transition to the diet, both lean (L-MAINT) and obese (O-MAINT) cats were fed to maintain BW for a period of 4 weeks. Daily energy requirement was calculated as 100 kcal X kg BW^0.67^ using current BW for each L-MAINT cat, and 130 kcal X kg BW^0.4^ using ideal BW for each O-MAINT cat [31]. Ideal BW was calculated based on BCS and current BW [32]. For obese cats with a BCS of 9/9, morphometry was used to determine body fat and then ideal BW was calculated [33]. Each cat’s BW, BCS and muscle condition score (MCS) [34] were re-assessed after 2 weeks and at the end of the 4-week maintenance period. For the lean cats, this was the end of the study, while the obese cats continued with energy restriction for weight loss for a period of 10 weeks (O-RESTRICT). Energy provision for weight loss was determined as 0.6 × (130 kcal × kg BW^0.4^) using ideal BW for each O-RESTRICT cat in the obese group [31, 35]. Assessments of BW, BCS and MCS were performed every other week for the duration of the 10-week restriction period. Acceptable weekly weight loss was considered to be 0.5–2% of initial BW and food amount was adjusted if these targets were not being met [36, 37].

### Laboratory analysis of blood samples

After an overnight fast, blood was collected from each cat via jugular or cephalic venipuncture at the end of the 4-week maintenance period for the lean and obese cats (L-MAINT, O-MAINT) and at the end of the 10-week restriction period for the obese cats (O-RESTRICT). Blood was centrifuged for 10 min at 3000 rpm after refrigeration at 4°C for 2 hours and serum removed and immediately submitted to the Animal Health Laboratory at the Ontario Veterinary College, Guelph, Canada and analyzed for glucose, cholesterol, high-density lipoprotein cholesterol (HDL-C), non-esterified fatty acids (NEFA), and triglycerides (TG) via photometry using a Roche Cobas 6000 c501 analyzer (Roche Diagnostics, Basel, Switzerland). Remaining samples were stored at -20°C until further analysis. Serum insulin was analyzed by Insulin ELISA Feline Kit (10–1233091; Mercodia, Alberta, Canada), previously validated for cats [38]. Samples were submitted to The Metabolomics Innovation Centre at the University of Alberta, Edmonton, Canada for serum metabolomics. First, quantitative nuclear magnetic resonance (NMR) spectroscopy, which is the targeted analysis of water-soluble metabolite classes including alcohols, amines, AA, organic acids, short-chain fatty acids, sugars and TCA cycle intermediates, was performed [39]. Second, direct infusion mass spectrometry (DI-MS) was conducted, targeting acylcarnitines, AA, biogenic amines, phospholipids and sphingolipids [40]. A total of 40 serum metabolites were analyzed using quantitative NMR spectroscopy and 150 metabolites using DI-MS. The 150 DI-MS metabolites were classified using the Human Metabolome Database [41]: acylcarnitines, AA, biogenic amines, glycerophospholipids (diacyl phosphatidylcholine and acyl-akyl phosphatidylcholine), LPCs, and sphingolipids and the total concentration for each group was calculated. Total acylcarnitines, total short-chain acylcarnitine (Sum of C2, C3, C4, C5, C5:1) and total long-chain acylcarnitines (Sum of C14 through C18) were also calculated for each group.

### Statistical analysis

Statistical analysis was performed using SAS University Edition 2, SAS Studio 3.8 (SAS Campus Drive, Cary, North Carolina, USA). Residuals were assessed for normal distribution using a Shapiro-Wilks test. A log transformation was performed on data that was not normal. For normally distributed data, a two-sided t-test was used to determine differences in concentration of each individual metabolite as well as total for each class between L-MAINT and O-MAINT and between L-MAINT and O-RESTRICT. A paired t-test was used to determine differences in individual metabolite concentrations and totals for each class between O-MAINT and O-RESTRICT. For non-parametric data, a Wilcoxon Signed-Rank test was used. Data is expressed as mean ± SE for normally distributed data and as median (minimum-maximum) for non-parametric data. A p-value of less than 0.05 was considered significant. Multivariate analysis, specifically principal component analysis (PCA) was done using Metaboanalyst 4.0 for NMR and DI-MS data sets [42]. The PCA analysis was performed using the prcomp package. Additionally, Metaboanalyst 4.0 was used to perform clustering analysis for each data set. Hierarchical clustering was performed and heatmaps were generated using Euclidean distance measures and clustered via the Ward algorithm, to visualize metabolite patterns and large-scale differences between groups.

## Results

### Energy intake and weight loss

All cats remained clinically healthy for the duration of the study period. Average daily energy intake for L-MAINT was 272.3 ± 46.5 kcals per day. Average daily energy intake for O-MAINT was 221.5 ± 24.4 kcals per day and for O-RESTRICT this was reduced to 138.2 ± 10.2 kcals per day. Average BW remained stable over the four weeks and at the end of the adaptation period was 4.45 ± 0.83 kg for L-MAINT and 6.97 ± 1.36 kg for O-MAINT [29]. At the end of the restriction period, average BW of O-RESTRICT was 6.3 ± 1.13 kg. Obese cats lost a total average of 672 ± 303 g over the 10-week period which is an average weight loss rate of 0.94 ± 0.28% of initial BW per week. Final BCS ranged from 6 to 9/9 and none of the cats reached their ideal BW by the end of the 10-week restriction period.

### Biochemical parameters

No differences were noted between groups in serum concentrations of glucose, cholesterol, HDL-C and NEFA (P>0.05) (Table 1). Serum TG concentrations were greater in O-MAINT compared to L-MAINT (P = 0.03). Serum insulin concentrations were lower in O-RESTRICT compared to O-MAINT (P = 0.01).

**Table 1:** Serum biochemical parameters from 14 lean (L-MAINT) and 16 obese cats during weight maintenance (O-MAINT) and after 10-week energy restriction (O-RESTRICT).

Parameter	L-MAINT (n = 14)	O-MAINT (n = 16)	O-RESTRICT (n = 16)	P-Value		
				L-MAINT: O-MAINT	L-MAINT: O-RESTRICT	O-MAINT: O-RESTRICT
Glucose (mmol/L)	4.80 (3.00–7.80)	4.80 (3.40–9.90)	4.95 (3.90–10.20)	0.77	0.86	0.70
Cholesterol (mmol/L)	71.25 ± 4.60	68.34 ± 3.95	67.53 ± 14.6163	0.65	0.58	0.79
HDLC (mmol/L)	69.84 (42.30–91.44)	60.39 (34.74–95.40)	70.83 (41.76–82.62)	0.25	0.36	0.62
NEFA (mmol/L)	0.61 ± 0.07	0.72 ± 0.08	0.72 ± 0.2342	0.46	0.19	0.98
**TG (mmol/L)**	**9.00 (9.40–19.80)** ^**A**^	**11.70 (7.20–86.40)** ^**B**^	9.90 (7.20–43.20) ^AB^	**0.03**	0.40	0.10
**Insulin (ng/L)**	231.46 (53.36–470.87) ^AB^	**285.44 (115.95–1579.22)** ^**A**^	**154.51 (82.91–519.29)** ^**B**^	0.31	0.17	**0.01**

Normally distributed data expressed as mean ± SE

Non-parametric data expressed as median (min-max).

Superscript capital letters (A, B) denote significant differences between groups with different letters where a p-value <0.05 is considered significant

No superscript letters in a row indicates no significant differences between groups for the measured parameter

HDLC, high density lipoprotein cholesterol; NEFA, non-esterified fatty acids; TG, triglycerides

### Direct infusion mass spectrometry

**Fig 1:**
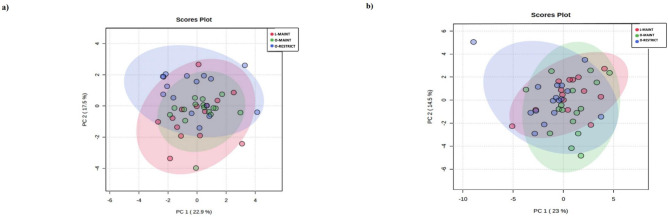
Scores plot between the selected principle components (PC) for nuclear magnetic resonance (NMR) spectroscopy (a) and direct infusion mass spectrometry (DI-MS) (b) data from 14 lean (L-MAINT) and 16 obese cats during weight maintenance (O-MAINT) and after 10-week energy restriction (O-RESTRICT). The explained variances are shown in brackets. Reprinted from Grant CE. Chapter 3. In Investigating the Effect of Energy Restriction for Weight loss in Obese Cats on Intake of Essential Nutrients and on Serum Metabolites [Thesis, University of Guelph] under a CC BY license, with permission from Caitlin Grant, original copyright 2020.

**Fig 2:**
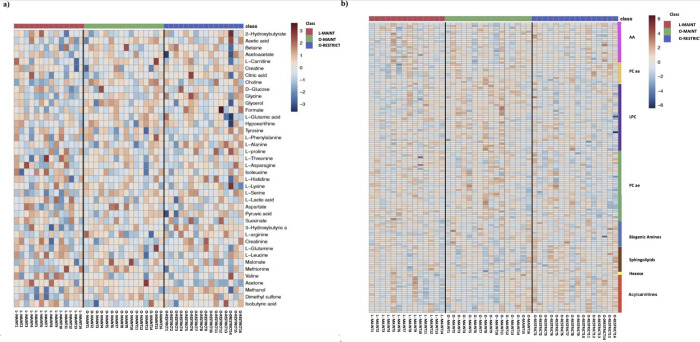
Clustering result shown as heatmap of nuclear magnetic resonance (NMR) spectroscopy data (a) and direct infusion mass spectrometry (DI-MS) (b) (distance measured using Euclidean) from 14 lean (L-MAINT) and 16 obese cats during weight maintenance (O-MAINT) and after 10-week energy restriction (O-RESTRICT). LPC = Lysophosphatidylcholines, PC aa = Phosphatidylcholine diacyl, PC ae = Phosphatidylcholine acyl-alkyl.

Figs 1 and 2 show the PCA and heatmap generated for the DI-MS data. Overall, the PCA shows a large degree of overlap between the three groups. Differences can be noted between groups on the heatmap and are described below.

#### Acylcarnitines

No differences were observed between L-MAINT and O-MAINT in any of the serum acylcarnitine concentrations (Table 2). However, a total of nine acylcarnitines had serum concentrations significantly greater in O-RESTRICT compared to O-MAINT including C14:1 (P = 0.01), C14:1OH (P = 0.01), C14:2 (P = 0.006), C16 (P = 0.02), C16:1 (P = 0.007), C18 (P = 0.03), C18:1 (P = 0.01), C18:1OH (P = 0.03), and C18:2 (P = 0.02). This is illustrated in the heatmap (Fig 2). Five of these, C14:1 (P = 0.04), C14:2 (P = 0.04), C16:1 (P = 0.01), C16:1 OH (P = 0.01) and C18:1 (P = 0.02) were also greater in O-RESTRICT than L-MAINT. Furthermore, when grouping acylcarnitines according to carbon length, O-RESTRICT had a greater concentration of long-chain acylcarnitines compared to O-MAINT (P = 0.04) and compared to L-MAINT (P = 0.03). Total short-chain acylcarnitines also differed with a higher number again in O-RESTRICT compared to O-MAINT (P = 0.02) and compared to L-MAINT (P = 0.049). Total serum acylcarnitine concentrations were similar between groups (P>0.05).

**Table 2:** Direct infusion mass spectrometry serum acylcarnitines (μM) from 14 lean (L-MAINT) and 16 obese cats during weight maintenance (O-MAINT) and after 10-week energy restriction (O-RESTRICT).

	Metabolite (μM)	L-MAINT (n = 14)	O-MAINT (n = 16)	O-RESTRICT (n = 16)	P-Value		
					L-MAINT: O-MAINT	L-MAINT: O-RESTRICT	O-MAINT: O-RESTRICT
Free	CO	25.80 (13.20–50.20)	28.90 (19.60–53.50)	29.00 (17.70–34.30)	0.40	0.69	0.62
Short	C2	2.38 (1.64–6.83)	2.84 (1.63–4.26)	3.33 (2.26–6.30)	0.62	0.06	0.06
	C3	0.15 ± 0.05	0.17 ± 0.06	0.16 ± 0.04	0.21	0.48	0.26
	C4	0.14 ± 0.03	0.15 ± 0.03	0.16 ± 0.04	0.47	0.21	0.43
	C5	0.18 ± 0.06	0.19 ± 0.04	0.19 ± 0.05	0.50	0.60	0.86
	C5:1	0.05 ± 0.01	0.04 ± 0.01	0.04 ± 0.01	0.29	0.06	0.21
Medium	C12	0.07 ± 0.004	0.07 ± 0.005	0.07 ± 0.003	0.73	0.67	0.98
Long	C14	0.11 (0.08–0.16)	0.11 (0.080–0.19)	0.12 (0.090–0.19)	0.66	0.38	0.38
	**C14:1**	**0.16 ± 0.01** ^**A**^	**0.16 ± 0.01** ^**A**^	**0.21 ± 0.02** ^**B**^	0.95	**0.04**	**0.01**
	**C14:1 OH**	0.030 ± 0.002^AB^	**0.029 ± 0.001** ^**A**^	**0.034 ± 0.002** ^**B**^	0.73	0.21	**0.01**
	**C14:2**	**0.024 (0.01–0.06)** ^**A**^	**0.024 (0.01–0.03)** ^**A**^	**0.033 (0.02–0.07)** ^**B**^	0.97	**0.04**	**0.006**
	**C14:2 OH**	0.021 ± 0.001	0.020 ± 0.001	0.020 ± 0.001	0.63	0.83	0.61
	**C16**	0.19 (0.10–0.30)^AB^	**0.18 (0.14–0.31)** ^**A**^	**0.24 (0.15–0.34)** ^**B**^	0.91	0.12	**0.02**
	**C16:1**	**0.073 (0.056–0.12)** ^**A**^	**0.070 (0.048–0.15)** ^**A**^	**0.10 (0.047–0.17)** ^**B**^	0.82	**0.01**	**0.007**
	**C16:1 OH**	**0.019 ± 0.001** ^**A**^	0.021 ± 0.001^AB^	**0.024 ± 0.001** ^**B**^	0.29	**0.01**	0.07
	C16:2	0.014 (0.008–0.039)	0.016 (0.010–0.023)	0.02 (0.0090–0.037)	0.51	0.21	0.21
	**C18**	0.098 (0.059–0.16)^AB^	**0.096 (0.059–0.15)** ^**A**^	**0.13 (0.078–0.17)** ^**B**^	0.63	0.16	**0.03**
	**C18:1**	**0.18 (0.095–0.34)** ^**A**^	**0.19 (0.10–0.48)** ^**A**^	**0.25 (0.12–0.46)** ^**B**^	0.47	**0.02**	**0.01**
	**C18:1 OH**	**0.022 ± 0.001** ^**A**^	**0.023 ± 0.001** ^**A**^	**0.026 ± 0.002** ^**B**^	0.66	0.05	**0.03**
	C18:2	0.070 (0.05–0.16)^AB^	**0.076 (0.04–0.16)** ^**A**^	**0.11 (0.04–0.18)** ^**B**^	0.73	0.10	**0.02**
**Total**	**Short chain Acylcarnitines**	**2.87 (2.06–7.63)** ^**A**^	**3.46 (1.97–4.82)** ^**A**^	**3.81 (2.76–6.81)** ^**B**^	0.34	0.049	**0.02**
	**Long chain Acylcarnitines**	**1.05 ± 0.08** ^**A**^	**1.09 ± 0.07** ^**A**^	**1.32 ± 0.09** ^**B**^	0.75	**0.03**	**0.04**
	Acylcarnitines	32.36 ± 2.92	35.36 ± 2.21	33.46 ± 1.64	0.41	0.74	0.82

Normally distributed data expressed as mean ± SE

Non-parametric data expressed as median (min-max).

Superscript capital letters (A, B) denote significant differences between groups with different letters where a p-value <0.05 is considered significant

No superscript letters in a row indicates no significant differences between groups for the measured parameter

Short-chain Acylcarnitines = C2+C3+C4+C5+C5:1

Long-chain Acylcarnitines = C14+C14:1+C14:1OH+C14:2+C14:2OH+C16+C16:1 = C16:1OH+C16:2+C18+C18:1+C18:1OH+C18:2

#### Amino acids

Four gluconeogenic AA, including alanine (P = 0.004), asparagine (P = 0.04), glutamine (P = 0.02) and glycine (P = 0.04), were greater in O-RESTRICT compared to O-MAINT (Table 3). Serum concentrations of glycine (P<0.001) and serine (P = 0.003) were also greater in O-RESTRICT compared to L-MAINT. At last, O-RESTRICT cats had greater total serum AA concentrations compared to O-MAINT (P = 0.02). Serum concentrations of serine were greater in O-MAINT compared to L-MAINT (P = 0.01); however, no other differences were noted between O-MAINT and L-MAINT or between O-RESTRICT and L-MAINT (P>0.05). Total AA concentration was not used in the heatmap (Fig 2); however, overall concentrations were greater in O-RESTRICT compared to O-MAINT (P = 0.02) (Table 3).

**Table 3:** Direct infusion mass spectrometry serum amino acids (μM) from 14 lean (L-MAINT) and 16 obese (O-MAINT) cats during weight maintenance and after 10-week energy restriction (O-RESTRICT).

Metabolite (μM)	L-MAINT (n = 14)	O-MAINT (n = 16)	O-RESTRICT (n = 16)	P-Value		
				L-MAINT: O-MAINT	L-MAINT: O-RESTRICT	O-MAINT: O-RESTRICT
**Alanine**	471.07 ± 25.48^AB^	**450.19 ± 29.10** ^**A**^	**565 .75± 39.79** ^**B**^	0.60	0.07	**0.004**
Arginine	52.50 (40.0–104.0)	54.50 (34.0–104.0)	60.50 (41.0–104.0)	0.87	0.32	0.64
**Asparagine**	74.15 (36.0–143.0)^AB^	**74.15 (37.0–96.0)** ^**A**^	**81.95 (60.7–94.5)** ^**B**^	0.93	0.87	**0.04**
Aspartic Acid	21.03 ± 2.27	23.67 ± 1.50	21.36 ± 1.26	0.33	0.90	0.22
**Glutamine**	725.00 (600.0–1040.0)^AB^	**691.00 (523.0–1050.0)** ^**A**^	**817.50 (650.0–974.0)** ^**B**^	0.39	0.25	**0.02**
Glutamic Acid	52.00 (20.9–97.5)	54.00 (31.3–86.9)	50.7 (19.9–128.0)	0.68	0.55	0.54
**Glycine**	**266.64 ± 17.63** ^**A**^	**300.50 ± 13.56** ^**A**^	**345.13 ± 14.16** ^**B**^	0.13	**<0.001**	**0.04**
Histidine	133.50 (106.0–167.0)	137.00 (91.0–157.0)	141.00 (115.0–165.0)	0.98	0.26	0.051
Isoleucine	60.66 ± 5.01	59.37 ± 2.59	59.53 ± 3.13	0.82	0.85	0.96
Leucine	119.00 (82.0–170.0)	111.00 (67.1–136.0)	113.00 (92.0–220.0)	0.20	0.62	0.79
Lysine	103.50 (69.0–157.0)	99.45 (76.0–149.0)	97.00 (53.5–308.0)	0.96	0.51	0.49
Methionine	64.51 ± 8.94	63.20 ± 5.53	54.78 ± 4.32	0.90	0.34	0.15
Ornithine	13.63 ± 0.70	12.73 ± 0.79	12.90 ± 0.86	0.41	0.53	0.85
Phenylalanine	79.56 ± 3.90	81.06 ± 2.53	77.15 ± 2.26	0.74	0.59	0.23
Proline	147.50 (69.0–434.0)	158.00 (92.0–259.0)	162.00 (97.0–538.0)	0.70	0.53	0.44
**Serine**	**135.88 ± 12.85** ^**A**^	**181.50 ± 11.26** ^**B**^	**189.81 ± 10.24** ^**B**^	**0.01**	**0.003**	0.50
Threonine	141.51 ± 15.09	131.06 ± 6	132.13 ± 5.87	0.53	0.57	0.91
Tyrosine	55.95 (37.6–70.6)	56.20 (29.7–69.3)	49.95 (29.0–96.7)	0.99	0.52	0.32
Valine	177.00 (104.0–256.0)	156.00 (101.0–207.0)	164.00 (137.0–413.0)	0.20	0.56	0.49
**Total Amino Acids**	2909.95 (2314.2–3962.5)^AB^	**2963.02 (2276.3–3617.4)** ^**A**^	**3152.70 (2841.0–4601.4)** ^**B**^	0.82	0.06	**0.02**

Normally distributed data expressed as mean ± SE

Non-parametric data expressed as median (min-max).

Superscript capital letters (A, B) denote significant differences between groups with different letters where a p-value <0.05 is considered significant

No superscript letters in a row indicates no significant differences between groups for the measured parameter

#### Biogenic amines

Biogenic amines are presented in Table 4. Serum histamine concentrations were greater in O-MAINT compared to L-MAINT (P = 0.04), while serum creatinine concentrations were greater in O-RESTRICT compared to O-MAINT (P = 0.02). No differences were observed between O-RESTRICT and L-MAINT for the specific biogenic amines (P>0.05). Also, the total serum biogenic amine concentrations were similar between groups (P>0.05).

**Table 4:** Direct infusion mass spectrometry serum biogenic amines (μM) from 14 lean (L-MAINT) and 16 obese (O-MAINT) cats during weight maintenance and after 10-week energy restriction (O-RESTRICT).

Metabolite (μM)	L-MAINT (n = 14)	O-MAINT (n = 16)	O-RESTRICT (n = 16)	P-Value		
				L-MAINT: O-MAINT	L-MAINT: O-RESTRICT	O-MAINT: O-RESTRICT
Acetylornithine	6.02 (4.06–9.68)	6.85 (5.46–9.33)	6.30 (4.33–9.85)	0.10	0.80	0.07
Asymmetric dimethylarginine	2.49 ± 0.15	2.58 ± 0.13	2.61 ± 0.12	0.62	0.52	0.86
Alpha-Aminoadipic acid	2.13 ± 0.18	1.98 ± 0.07	2.10 ± 0.11	0.43	0.90	0.32
Carnosine	46.73 ± 3.48	43.12 ± 3.26	42.69 ± 2.79	0.46	0.37	0.86
**Creatinine**	128.72 ± 6.92^AB^	**113.68 ± 5.40** ^**A**^	**133.63 ± 7.15** ^**B**^	0.09	0.62	**0.02**
**Histamine**	**0.046 (0.04–0.07)** ^**A**^	**0.052 (0.04–0.1)** ^**B**^	0.050 (0.04–0.1)^AB^	**0.04**	0.38	0.27
Kynurenine	8.46 (4.51–15.20)	8.27 (4.85–23.90)	8.36 (6.53–14.60)	0.76	0.69	0.97
Methioninesulfoxide	4.99 (2.24–16.10)	8.10 (3.33–14.80)	6.33 (2.14–23.50)	0.17	0.53	0.83
Trans-hydroxyproline	30.67 ± 3.83	36.92 ± 3.38	36.23 ± 3.20	0.23	0.27	0.81
Putrescine	1.07 ± 0.08	1.17 ± 0.10	1.09 ± 0.092	0.48	0.91	0.47
Serotonin	12.80 ± 0.63	13.05 ± 0.97	13.07 ± 0.95	0.83	0.82	0.99
Spermidine	0.6 (0.42–1.25)	0.64 (0.39–0,99)	0.51 (0.36–1.02)	0.37	0.13	0.26
Spermine	0.28 ± 0.02	0.30 ± 0.03	0.33 ± 0.04	0.76	0.42	0.35
Taurine	226.50 (129.0–302.0)	222.00 (120.0–293.0)	208.50 (142.0–297.0)	0.66	0.94	0.97
Total Biogenic Amines	459.32 ± 92.98	445.66 ± 63.88	463.85 ± 62.77	0.64	0.88	0.26

Normally distributed data expressed as mean ± SE

Non-parametric data expressed as median (min-max).

Superscript capital letters (A, B) denote significant differences between groups with different letters where a p-value <0.05 is considered significant

No superscript letters in a row indicates no significant differences between groups for the measured parameter

#### Glycerophospholipids–phosphatidylcholine diacyl

Serum concentrations of phosphatidylcholine diacyl (PC aa) C38:5 and PC aa C32:1 were greater in O-MAINT compared to L-MAINT (P = 0.04 and 0.04, respectively) (Table 5). Nine metabolites of this family also had lower concentrations in O-RESTRICT compared to O-MAINT including PC aa C24:0 (P = 0.04), PC aa C32:2 (P = 0.007), PC aa C32:3 (P = 0.049), PC aa C34:4 (P = 0.009), PC aa C36:4 (P = 0.03), PC aa C36:5 (P = 0.007), PC aa C36:6 (P = 0.001), PC aa PC aa C38:4 (P = 0.01), and PC aa C38:5 (P = 0.045).This is visualized in the heatmap (Fig 2). Between O-RESTRICT and L-MAINT, only PC aa C42:4 was greater in L-MAINT (P = 0.03). Total serum PC aa concentrations did not differ between groups (P>0.05).

**Table 5:** Direct infusion mass spectrometry serum phosphatidylcholine diacyls (PC aa) (μM) from 14 lean (L-MAINT) and 16 obese (O-MAINT) cats during weight maintenance and after 10-week energy restriction (O-RESTRICT).

Metabolite (μM)	L-MAINT (n = 14)	O-MAINT (n = 16)	O-RESTRICT (n = 16)	P-Value		
				L-MAINT: O-MAINT	L-MAINT: O-RESTRICT	O-MAINT: O-RESTRICT
**PC aa C24:0**	0.11 ± 0.01^AB^	**0.13 ± 0.01** ^**A**^	**0.11 ± 0.01** ^**B**^	0.14	0.81	**0.04**
PC aa C28:1	2.12 (1.38–2.86)	2.16 (1.43–3.59)	1.90 (1.30–2.75)	0.60	0.36	0.22
PC aa C30:0	3.61 ± 0.29	4.02 ± 0.30	3.35 ± 0.25	0.33	0.24	0.06
PC aa C32:0	6.41 ± 0.36	6.93 ± 0.42	6.31 ± 0.34	0.21	0.37	0.85
**PC aa C32:1**	**7.39 ± 0.57** ^**A**^	**9.38 ± 0.71** ^**B**^	**7.95 ± 0.63** ^**AB**^	**0.04**	0.52	0.13
**PC aa C32:2**	1.21 (0.50–3.98)^AB^	**2.16 (0.75–3.05)** ^**A**^	**1.11 (0.22–2.70)** ^**B**^	0.06	0.63	**0.007**
**PC aa C32:3**	0.24 ± 0.03^AB^	**0.26 ± 0.03** ^**A**^	**0.19 ± 0.02** ^**B**^	0.61	0.16	**0.049**
PC aa C34:1	111.35 ± 4.89	119.54 ± 6.71	111.23 ± 4.64	0.34	0.99	0.21
PC aa C34:2	294.07 ± 22.70	335.75 ± 18.79	308.63 ± 15.81	0.17	0.60	0.23
PC aa C34:3	24.49 ± 2.69	28.54 ± 2.27	23.51 ± 1.92	0.26	0.77	0.06
**PC aa C34:4**	0.84 ± 0.10^AB^	**1.01 ± 0.09** ^**A**^	**0.73 ± 0.08** ^**B**^	0.20	0.40	**0.009**
PC aa C36:0	4.97 (3.08–7.52)	4.49 (3.07–9.63)	4.10 (2.66–6.63)	0.98	0.11	0.12
PC aa C36:1	102.50 (72.70–130.00)	110.50 (69.20–157.00)	96.45 (65.00–127.00)	0.35	0.48	0.13
PC aa C36:2	569.50 (411.00–707.00)	631.00 (458.00–840.00)	589.50 (409.00–808.00)	0.05	0.51	0.16
PC aa C36:3	148.79 ± 13.55	173.88 ± 11.07	149.67 ± 9.65	0.16	0.96	0.08
**PC aa C36:4**	106.53 ± 5.99^AB^	**121.64 ± 6.78** ^**A**^	**104.47 ± 5.07** ^**B**^	0.11	0.79	**0.03**
**PC aa C36:5**	13.88 ± 1.25^AB^	**15.17 ± 1.03** ^**A**^	**12.39 ± 0.83** ^**B**^	0.43	0.32	**0.007**
**PC aa C36:6**	0.60 ± 0.08^AB^	**0.63 ± 0.06** ^**A**^	**0.47 ± 0.06** ^**B**^	0.84	0.19	**0.001**
**PC aa C38:0**	1.36 ± 0.10	1.16 ± 0.10	1.09 ± 0.09	0.18	0.06	0.31
**PC aa C38:1**	2.05 (1.26–2.88)	1.74 (1.07–3.14)	1.56 (0.91–3.14)	0.23	0.12	0.64
**PC aa C38:3**	36.85 ± 1.66^AB^	**41.43 ± 2.64** ^**A**^	**34.99 ± 2.55** ^**B**^	0.17	0.52	0.07
**PC aa C38:4**	179.50 ± 12.54^AB^	**210.88± 14.74** ^**A**^	**188.13 ± 12.36** ^**B**^	0.12	0.63	**0.01**
**PC aa C38:5**	**43.62 ± 2.52** ^**A**^	**52.58 ± 3,20** ^**B**^	**48.19 ± 3.20** ^**A**^	**0.04**	0.28	**0.045**
**PC aa C38:6**	27.35 (18.20–53.60)	31.80 (19.30–73.90)	31.00 (17.10–66.80)	0.25	0.15	0.84
**PC aa C40:2**	0.79 ± 0.06	0.79 ± 0.06	0.68 ± 0.05	0.95	0.16	0.07
**PC aa C40:3**	1.20 (0.80–1.62)	1.27 (0.80–2.11)	0.93 (0.65–1.76)	0.70	0.14	0.14
**PC aa C40:4**	7.87 (5.40–9.78)^AB^	**7 .42 (5.01–14.10)** ^**A**^	**6.16 (3.90–12.00)** ^**B**^	0.49	0.07	0.07
**PC aa C40:5**	11.69 ± 0.88	15.10 ± 1.43	14.90 ± 1.40	0.06	0.07	0.77
**PC aa C40:6**	21.65 (9.86–59.90)	26.50 (11.50–97.00)	29.70 (13.80–80.00)	0.27	0.09	0.34
**PC aa C42:0**	0.06 ± 0.01	0.06 ± 0.01	0.07 ± 0.01	0.92	0.81	0.55
**PC aa C42:1**	0.11 (0.06–0.16)	0.10 (0.07–0.16)	0.09 (0.03–0.16)	0.16	0.07	0.56
**PC aa C42:2**	0.45 (0.14–0.80)	0.42 (0.27–0.73)	0.37 (0.28–0.63)	0.73	0.28	0.52
**PC aa C42:4**	**0.35 (0.15–0.45)** ^**A**^	0.28 (0.20–0.59)^AB^	**0.26 (0.14–0.48)** ^**B**^	0.34	**0.03**	0.11
**PC aa C42:5**	0.33 (0.21–0.45)	0.37 (0.21–0.61)	0.30 (0.21–0.67)	0.10	0.86	0.13
**PC aa C42:6**	0.54 (0.35–0.67)	0.58 (0.29–1.42)	0.55 (0.24–1.75)	0.44	0.92	0.42
**Total PC aa**	1723.01 (1245.25–2256.13)	1905.78 (1443.94–2561.36)	1725.79 (1346.93–2487.57)	0.05	0.60	0.12

Normally distributed data expressed as mean ± SE

Non-parametric data expressed as median (min-max).

Superscript capital letters (A, B) denote significant differences between groups with different letters where a p-value <0.05 is considered significant

No superscript letters in a row indicates no significant differences between groups for the measured parameter

#### Glycerophospholipids—phosphatidylcholine acyl-alkyl

There were no differences in any of the phosphatidylcholine acyl-alkyl (PC ae) metabolite concentrations between the L-MAINT and O-MAINT groups (P>0.05) (Table 6). Serum concentrations of three metabolites in this group were lower in O-RESTRICT compared to O-MAINT including PC ae C38:0 (P = 0.04), PC ae C40:1 (P = 0.01), and PC ae C42:0 (P = 0.04). Serum concentrations of PC ae C38:1 were lower in O-RESTRICT compared to L-MAINT (P = 0.03) as well as five other metabolites including PC ae C30:0 (P = 0.04), PC ae C40:3 (P = 0.02), PC ae C42:4 (P = 0.03), PC ae C44:3 (P = 0.03) and PC ae C44:5 (P = 0.049). Total serum PC ae concentrations were similar between groups (P>0.05).

**Table 6:** Direct infusion mass spectrometry serum phosphatidylcholine acyl-alkyls (PC ae) (μM) from 14 lean (L-MAINT) and 16 obese (O-MAINT) cats during weight maintenance and after 10-week energy restriction (O-RESTRICT).

Metabolite (μM)	L-MAINT (n = 14)	O-MAINT (n = 16)	O-RESTRICT (n = 16)	P-Value		
				L-MAINT: O-MAINT	L-MAINT: O-RESTRICT	O-MAINT: O-RESTRICT
**PC ae C30:0**	**0.40 (0.16–0.61)** ^**A**^	0.34 (0.24–0.66)^AB^	**0.32 (0.22–0.48)** ^**B**^	0.37	**0.04**	0.11
PC ae C32:1	0.78 ± 0.05	0.80 ± 0.06	0.69 ± 0.04	0.81	0.18	0.10
PC ae C32:2	0.25 ± 0.02	0.26 ± 0.02	0.26 ± 0.02	0.59	0.68	0.82
PC ae C34:0	0.85 (0.48–2.30)	0.68 (0.49–1.50)	0.67 (0.45–0.95)	0.26	0.05	0.44
PC ae C34:1	8.39 (4.82–11.70)	7.34 (5.14–12.50)	7.75 (4.80–9.75)	0.94	0.53	0.76
PC ae C34:2	8.97 (4.95–11.80)	7.18 (5.74–13.40)	8.51 (5.35–12.60)	0.20	0.53	0.13
PC ae C34:3	2.54 (1.30–2.79)	1.84 (1.35–3.65)	1.93 (1.38–3.27)	0.08	0.47	0.41
PC ae C36:0	1.66 (0.62–4.53)	1.29 (0.83–2.48)	1.25 (0.75–2.56)	0.28	0.09	0.47
PC ae C36:1	6.65 (4.00–7.79)	5.41 (4.14–11.90)	6 (4–7)	0.79	0.09	0.30
PC ae C36:2	17.10 ± 1.20	18.79 ± 1.32	16.95 ± 0.95	0.36	0.92	0.17
PC ae C36:3	5.78 (3.30–8.37)	4.86 (3.38–8.13)	5.25 (3.30–7.10)	0.59	0.28	0.77
PC ae C36:4	3.85 (2.16–5.93)	3.61 (2.16–5.81)	3.87 (2.16–4.81)	0.93	0.58	0.93
PC ae C36:5	1.60 ± 0.13	1.55 ± 0.13	1.62 ± 0.10	0.77	0.99	0.51
**PC ae C38:0**	2.86 ± 0.19^AB^	**3.02 ± 0.19** ^**A**^	**2.65 ± 0.19** ^**B**^	0.57	0.43	**0.004**
**PC ae C38:1**	**3.30 (1.56–4.49)** ^**A**^	**2.78 (1.64–7.77)** ^**A**^	**2.54 (1.27–4.56)** ^**B**^	0.67	**0.03**	0.07
PC ae C38:2	14.40 (6.55–18.00)	11.75 (8.09–28.00)	12.00 (5.71–21.20)	0.74	0.10	0.14
PC ae C38:3	5.73 (2.46–7.57)	4.89 (3.04–10.50)	4.64 (2.53–7.61)	0.93	0.17	0.26
PC ae C38:4	7.97 (5.10–14.30)	9.21 (5.50–14.80)	8.22 (5.03–11.50)	0.65	0.74	0.17
PC ae C38:5	4.53 ± 0.32	4.82 ± 0.36	4.80 ± 0.28	0.56	0.53	0.94
PC ae C38:6	1.84 ± 0.17	1.64 ± 0.16	1.76 ± 0.17	0.39	0.74	0.27
**PC ae C40:1**	**1.59± 0.10** ^**AB**^	**1.67 ± 0.14** ^**A**^	**1.44 ± 0.11** ^**B**^	0.66	0.34	**0.01**
PC ae C40:2	1.31 (0.60–1.42)	1.17 (0.76–1.95)	1.12 (0.68–1.68)	0.44	0.44	0.35
**PC ae C40:3**	**1.42 (0.69–1.66)** ^**A**^	1.11 (0.71–2.86)^AB^	**1.00 (0.68–2.01)** ^**B**^	0.38	**0.02**	0.15
PC ae C40:4	4.85 (3.06–6.95)	4.58 (2.72–9.79)	4.22 (2.24–6.87)	0.88	0.06	0.14
PC ae C40:5	2.23. ± 0.18	2.28 ± 0.17	2.14 ± 0.15	0.88	0.73	0.26
PC ae C40:6	1.81 ± 0.12	1.28 ± 0.15	1.40 ± 0.15	0.60	0.35	0.16
**PC ae C42:0**	0.86 (0.61–1.08)^AB^	**0.90 (0.59–1.80)** ^**A**^	**0.88 (0.49–1.83)** ^**B**^	0.23	0.88	**0.04**
PC ae C42:1	0.81 (0.54–1.09)	0.89 (0.52–1.90)	0.82 (0.44–2.07)	0.21	0.64	0.31
PC ae C42:2	0.5 3(0.36–0.69)	0.56 (0.25–0.82)	0.45 (0.23–0.87)	0.92	0.40	0.41
PC ae C42:3	0.44 (0.27–0.84)	0.43 (0.19–0.97)	0.45 (0.16–0.91)	0.80	0.60	0.86
PC ae C42:4	0.32 (0.19–0.49)^A^	0.27 (0.13–0.68)^AB^	0.23 (0.10–0.48)^B^	0.41	**0.03**	0.26
**PC ae C44:3**	0.067 (0.03–0.10)^A^	0.047 (0.03–0.09)^AB^	0.050 (0.03–0.07)^B^	0.18	**0.03**	0.62
PC ae C44:4	0.092 (0.05–0.12)	0.076 (0.06–0.12)	0.067 (0.05–0.13)	0.21	0.06	0.31
**PC ae C44:5**	**0.088 ± 0.01** ^**A**^	0.084 ± 0.01^AB^	**0.073 ± 0.00** ^**B**^	0.67	**0.049**	0.16
PC ae C44:6	0.078 ± 0.01	0.079 ± 0.01	0.070 ± 0.01	0.93	0.38	0.26
Total PC ae	122.40 (63.36–140.49)	106.24 (75.36–183.61)	106.61 (71.82–143.31)	0.89	0.28	0.36

Normally distributed data expressed as mean ± SE.

Non-parametric data expressed as median (min-max).

Superscript capital letters (A, B) denote significant differences between groups with different letters where a p-value <0.05 is considered significant.

No superscript letters in a row indicates no significant differences between groups for the measured parameter

#### Lysophospholipids–lysophosphatidylcholines

Serum concentration of two metabolites in the LPC family were greater in O-MAINT compared to L-MAINT including Lysophosphatidylcholines acyl (LPC a) C16:1 (P = 0.02) and LPC a C20:4 (P = 0.04) (Table 7). Serum concentrations of LPC a C28:1 were lower in O-RESTRICT compared to O-MAINT (P = 0.005). No differences were observed between O-RESTRICT and L-MAINT and also total serum LPC concentrations were similar between groups (P>0.05).

**Table 7:** Direct infusion mass spectrometry serum lysophosphatidylcholines (LPC) (μM) from 14 lean (L-MAINT) and 16 obese (O-MAINT) cats during weight maintenance and during 10-week energy restriction (O-RESTRICT).

Metabolite (μM)	L-MAINT (n = 14)	O-MAINT (n = 16)	O-RESTRICT (n = 16)	P-Value		
				L-MAINT: O-MAINT	L-MAINT: O-RESTRICT	O-MAINT: O-RESTRICT
LPC C14:0	6.64 ± 0.16	6.87 ± 0.15	6.83 ± 0.14	0.30	0.36	0.75
LPC C16:0	66.74 ± 4.38	68.66 ± 3.20	69.63 ± 3.48	0.72	0.60	0.82
**LPC C16:1**	**2.44 ± 0.24** ^**A**^	**3.30 ± 0.26** ^**B**^	3.23 ± 0.34^AB^	**0.02**	0.08	0.77
LPC C17:0	1.47 ± 0.09	1.57 ± 0.10	1.52 ± 0.10	0.49	0.74	0.66
LPC C18:0	52.05 ± 2.96	54.92 ± 2.87	54.34 ± 2.75	0.49	0.58	0.82
LPC C18:1	21.81 ± 1.72	23.71 ± 1.26	23.78 ± 2.01	0.37	0.47	0.98
LPC C18:2	3.05 (20.8–79.00)	49.35 (27.90–72.70)	44.55 (27.9–122.00)	0.10	0.30	0.97
LPC C20:3	1.36 ± 0.12	1.52 ± 0.13	1.37 ± 0.15	0.40	0.97	0.40
**LPC C20:4**	**5.15 ± 0.31** ^**A**^	**6.23 ± 0.39** ^**B**^	5.87 ± 0.43^AB^	**0.04**	0.19	0.44
LPC C28:0	0.38 ± 0.03	0.41 ± 0.03	0.35 ± 0.04	0.52	0.31	0.11
**LPC C28:1**	0.77 ± 0.05^AB^	**0.89 ± 0.07** ^**A**^	**0.68 ± 0.06** ^**B**^	0.19	0.18	**0.005**
Total LPC	203.04 (133.06–292.78)	202.84 (170.98–300.35)	204.50 (153.32–356.29)	0.34	0.55	0.98

Normally distributed data expressed as mean ± SE

Non-parametric data expressed as median (min-max).

Superscript capital letters (A, B) denote significant differences between groups with different letters where a p-value <0.05 is considered significant

No superscript letters in a row indicates no significant differences between groups for the measured parameter

#### Sphingolipids

There were no differences for any sphingolipid between L-MAINT and O-MAINT or between L-MAINT and O-RESTRICT (P>0.05) (Table 8). Serum concentrations of five sphingolipids were greater in O-RESTRICT compared to O-MAINT including hydroxysphingomyeline C16:1 (P = 0.007), sphingomyeline C16:1 (P = 0.01), sphingomyeline C18:0 (P = 0.03), sphingomyeline C18:1 (P = 0.0007) and sphingomyeline C26:1 (P = 0.03). Total serum sphingolipid concentrations were similar between groups (P>0.05).

**Table 8:** Direct infusion mass spectrometry serum sphingolipids (μM) from 14 lean (L-MAINT) and 16 obese (O-MAINT) cats during weight maintenance and during 10-week energy restriction (O-RESTRICT).

Metabolite (μM)	L-MAINT (n = 14)	O-MAINT (n = 16)	O-RESTRICT (n = 16)	P-Value		
				L-MAINT: O-MAINT	L-MAINT: O-RESTRICT	O-MAINT: O-RESTRICT
Hydroxysphingomyeline C14:1	11.44 ± 0.56	11.53 ± 0.84	11.91 ± 0.56	0.93	0.55	0.52
**Hydroxysphingomyeline C16:1**	2.97 ± 0.17^AB^	**2.73 ± 0.20** ^**A**^	**3.14 ± 0.15** ^**B**^	0.37	0.46	**0.007**
Hydroxysphingomyeline C22:1	15.82 ± 0.88	14.39 ± 1.03	14.86 ± 0.59	0.31	0.36	0.57
Hydroxysphingomyeline C22:2	6.49 ± 0.29	6.02 ± 0.51	6.56 ± 0.38	0.42	0.89	0.16
Hydroxysphingomyeline C24:1	1.85 ± 0.08	1.85 ± 0.13	2.07 ± 0.10	0.99	0.10	0.08
Sphingomyeline C16:0	113.50 (66.50–144.00)	99.75 (64.60–157.00)	108.00 (79.90–143.00)	0.95	0.69	0.51
**Sphingomyeline C16:1**	5.95 ± 0.29^AB^	**6.10 ± 0.39** ^**A**^	**6.89 ± 0.37** ^**B**^	0.75	0.06	**0.01**
**Sphingomyeline C18:0**	17.54 ± 0.97^AB^	**15.88 ± 1.12** ^**A**^	**18.86 ± 0.86** ^**B**^	0.28	0.32	**0.03**
**Sphingomyeline C18:1**	3.62 ± 0.23^AB^	**3.37 ± 0.27** ^**A**^	**4.22 ± 0.25** ^**B**^	0.50	0.10	**0.0007**
Sphingomyeline C20:2	0.42 (0.25–0.60)	0.42 (0.23–0.74)	0.42 (0.24–0.56)	0.56	0.96	0.66
Sphingomyeline C24:0	20.10 (12.40–24.60)	17.80 (14.40–28.50)	17.50 (14.40–22.80)	0.76	0.23	0.43
Sphingomyeline C24:1	25.95 ± 1.43	25.40 ± 1.44	27.19 ± 1.04	0.79	0.48	0.20
Sphingomyeline C26:0	0.35 ± 0.03	0.36 ± 0.02	0.36 ± 0.02	0.25	0.90	0.05
**Sphingomyeline C26:1**	0.31 ± 0.03^AB^	**0.26 ± 0.03** ^**A**^	**0.34 ± 0.02** ^**B**^	0.36	0.41	**0.03**
Total Sphingolipids	228.90 (145.57–289.07)	196.52 (137.04–310.68)	224.43 (165.24–291.62)	0.74	0.70	0.35

Normally distributed data expressed as mean ± SE. Non-parametric data expressed as median (min-max).

Superscript capital letters (A, B) denote significant differences between groups with different letters where a p-value <0.05 is considered significant

No superscript letters in a row indicates no significant differences between groups for the measured parameter

#### Miscellaneous/tricarboxylic acid cycle metabolites

For both hexose and citrate, there was no difference between L-MAINT and O-MAINT or between L-MAINT and O-RESTRICT (P>0.05). The serum concentrations of hexose in O-RESTRICT were greater than O-MAINT (P = 0.03), while the serum concentrations of citrate in O-RESTRICT were lower than O-MAINT (P = 0.04) (Table 9).

**Table 9:** Direct infusion mass spectrometry serum miscellaneous/tricarboxylic acid cycle metabolites (μM) from 14 lean (L-MAINT) and 16 obese (O-MAINT) cats during weight maintenance and during 10-week energy restriction (O-RESTRICT).

Metabolite (μM)	L-MAINT (n = 14)	O-MAINT (n = 16)	O-RESTRICT (n = 16)	P-Value		
				L-MAINT: O-MAINT	L-MAINT: O-RESTRICT	O-MAINT: O-RESTRICT
**Hexose**	5661.50 (4026.00–9003.00) ^AB^	**5376.00 (4440.00–10896.00)** ^**A**^	**6354.00 (4690.00–10890.00)** ^**B**^	0.98	0.11	**0.03**
**Citrate**	20.69 ± 1.63 ^AB^	**21.30 ± 1.96** ^**A**^	**17.37 ± 1.31** ^**B**^	0.82	0.11	**0.04**

Normally distributed data expressed as means ± SE

Non-normally distributed data expressed as median + range

Capital letters (A, B) denote significant differences between groups with different letters where a p-value <0.05 is considered significant

### Quantitative nuclear magnetic resonance spectroscopy

Results of the NMR analysis are viewed in the generated PCA (Fig 1) and heatmap (Fig 2) images. Overall, PCA image illustrates similarities and differences among the three groups. Serum l-serine, glycerol and 3 hydroxybutyric acid concentrations were greater (P = 0.0045, 0.03, & 0.03) in O-MAINT compared to L-MAINT. (Table 10). The O-RESTRICT group had nine metabolites with greater serum concentrations than O-MAINT and included glycine (P = 0.01), l-alanine (P = 0.004), l-glutamine (P = 0.03), l-histidine (P = 0.004), 2-hydroxybutyrate (P = 0.02), isobutryric acid (P = 0.03), creatinine (P = 0.01), citric acid (P = 0.04), and methanol (P = 0.02). Six metabolites had concentrations greater in O-RESTRICT compared to L-MAINT, including glycine (P = 0.002), l-alanine (P = 0.03), l-serine (P = 0.0009), 3-hydroxybutyric acid (P = 0.007), formate (P = 0.03), and methanol (P = 0.02). Lower serum acetone concentrations were observed in O-RESTRICT than in O-MAINT (P = 0.03) and L-MAINT (P = 0.02).

**Table 10:** Quantitative nuclear magnetic resonance spectroscopy of serum metabolites (μM) from 14 lean (L-MAINT) and 16 obese cats during weight maintenance (O-MAINT) and after 10-week energy restriction (O-RESTRICT).

Metabolite (μM)	L-MAINT (n = 14)	O-MAINT (n = 16)	O-RESTRICT (n = 16)	P-Value		
				L-MAINT: O-MAINT	L-MAINT: O-RESTRICT	O-MAINT: O-RESTRICT
**Gluconeogenic Amino Acids**						
Aspartate	20.31 (9.63–39.25)	20.94 (16.38–37.50)	21.00 (13.38–44.25)	0.73	0.81	0.61
**Glycine**	**268.34 ± 18.56** ^**A**^	**294.07 ± 12** ^**A**^	**344.55 ± 13.37** ^**B**^	0.24	**0.002**	**0.01**
**l-Alanine**	**453 ± 24.11** ^**A**^	**442.41 ± 28** ^**A**^	**562.88 ± 41.98** ^**B**^	0.78	**0.03**	**0.004**
l-Arginine	51.81 (34.13–98.38)	51.00 (31.75–93.63)	57.94 (40.75–99.00)	0.90	0.13	0.23
l-Asparagine	72.69 (43.25–138.63)	72.56 (45.63–92.25)	80.94 (57.13–96.88)	0.53	0.76	0.06
l-Glutamic Acid	53.73 ± 5.24	50.49 ± 3.12	48.92 ± 5.16	0.59	0.52	0.81
**l-Glutamine**	**734.09 ± 29.16** ^**AB**^	**721.69 ± 25.98** ^**A**^	**799.91 ± 21.15** ^**B**^	0.75	0.07	**0.03**
**l-Histidine**	**125.56 ± 5.11** ^**AB**^	**123.07 ± 2.53** ^**A**^	**135.69 ± 3.60** ^**B**^	0.67	0.11	**0.004**
l-Proline	130 (67–413)	143.44 (102.88–242.13)	150.75 (101.38–549.13)	0.59	0.26	0.30
**l-Serine**	**132.04 ± 12.32** ^**A**^	**182.16 ± 10.70** ^**B**^	**192.20 ± 10.71** ^**B**^	**0.0045**	**0.0009**	0.45
Methionine	66.30 ± 9.02	65.29 ± 5.29	57.00 ± 4.57	0.78	0.56	0.16
Valine	168.25 (99.63–265.63)	165.25 (103.88–201.38)	167.94 (123.00–442.88)	0.23	0.75	0.63
**Ketogenic Amino Acids**						
l-Leucine	119.63 (79.00–174.00)	115.00 (67.50–134.75)	111.44 (84.25–233.50)	0.20	0.68	0.38
l-Lysine	102.93 (70.75–140.50)	95.13 (68.63–137.63)	103.00 (57.75–298.25)	0.81	0.81	0.71
**Gluconeogenic and Ketogenic Amino Acids**						
Isoleucine	55.63 (26.13–92.38)	57.06 (30.13–69.13)	54.50 (41.13–118.13)	0.60	0.91	0.56
l-Phenylalanine	73 (59–100)	80.38 (58.25–89.38)	76.25 (61.13–110.25)	0.82	0.55	0.78
l-Threonine	138.17 ± 15.49	128.94 ± 6.20	133.37 ± 6.22	0.59	0.81	0.64
Tyrosine	59.81 (34.13–63.63)	52.31 (27.38–61.88)	49.25 (38.38–106.63)	0.76	0.82	0.72
**Augmented Amino Acid Degradation Products**						
**2-Hydroxybutyrate**	**20.13 (9.00–34.00)** ^**AB**^	**19.00 (10.25–26.00)** ^**A**^	**20.38 (12.38–89.75)** ^**B**^	0.21	0.74	**0.02**
**Isobutryric Acid**	**13.25 (8.25–24.50)** ^**AB**^	**10.56 (7.38–20.88)** ^**B**^	**13.00 (8.00–22.00)** ^**A**^	0.08	0.77	**0.03**
**Creatinine**	**122.46 ± 5.47** ^**AB**^	**112.98 ± 4.68** ^**A**^	**132.64 ± 6.29** ^**B**^	0.20	0.24	**0.01**
**Glycolysis**						
Acetic Acid	6.63 (3.50–15.13)	8.13 (3.13–12.13)	7.13 (4.75–30.38)	0.20	0.42	0.69
D-Glucose	5434.25 (3823.38–8981.25)	5097.63 (4264.13–10676.50)	5824.00 (4447.50–10148.38)	0.98	0.19	0.13
l-Lactic Acid	3237.79 ± 374.25	2721.07 ± 171.53	3032.31 ± 258.95	0.23	0.65	0.19
**Lipolysis**						
**Glycerol**	**475.07 ± 20.71** ^**A**^	**570.12 ± 36.19** ^**B**^	**506.65 ±29.22** ^**AB**^	**0.03**	0.40	0.10
**Tricarboxylic Acid Cycle**						
**Citric Acid**	**173.88 (119.75–324.50)** ^**AB**^	**167.69 (118.50–278.13)** ^**A**^	**210.81 (144.63–308.88)** ^**B**^	0.59	0.10	**0.04**
Malonate	13.13 (5.75–17.50)	14.00 (8.63–31.13)	12.81 (8.75–23.25)	0.07	0.30	0.27
Pyruvic Acid	56.25 (17.38–187.63)	44.25 (11.63–149.75)	74.56 (2.63–172.88)	0.46	1.0	0.20
Succinate	3.93 ± 0.55	3.52 ± 0.30	4.03 ± 0.66	0.51	0.89	0.51
**Ketogenesis**						
**3-Hydroxybutyric Acid**	**23.19 (10.88–91.38)** ^**A**^	**33.38 (21.88–65.50)** ^**B**^	**41.06 (15.90–89.75)** ^**B**^	**0.03**	**0.007**	0.10
Acetoacetate	13.44 (7.75–20.50)	13.06 (10.25–27.25)	16.00 (4.00–25.38)	0.44	0.18	0.60
Acetone	**21.56 (3.25–167.75)** ^**A**^	**22.75 (14.88–215.50)** ^**A**^	**65.94 (3.75–74.13)** ^**B**^	0.86	**0.02**	**0.03**
**One Carbon Metabolism**						
Betaine	170.75 (102.50–326.00)	183.56 (131.75–354.13)	204.50 (147.13–358.75)	0.43	0.08	0.26
Choline	10.68 ± 1.30	13.36 ± 0.9	12.50 (7.00–19.88)	0.09	0.17	0.42
Creatine	7.88 (1.13–15.38)	5.88 (1.00–19.38)	9.81 (3.00–15.25)	0.45	0.28	0.07
**Formate**	**13.00 (10.38–21.50)** ^**A**^	**17.00 (12.00–24.63)** ^**AB**^	**16.31 (12.25–53.88)** ^**B**^	0.10	**0.03**	0.59
l-Carnitine	27.25 (14.13–51.75)	32.13 (21.50–54.38)	31.69 (19.38–43.13)	0.15	0.35	0.45
**Purine Degradation**						
Hypoxanthine	19.08 ± 1.36	20.82 ± 1.34	20.09 ± 1.26	0.37	0.59	0.66
**Other**						
Dimethylsulfone	10.69 (3.00–27.38)	16.06 (6.00–30.88)	16.94 (5.00–31.63)	0.27	0.17	0.78
Methanol	**50.31 (38.13–78.25)** ^**A**^	**61.75 (40.88–75.38)** ^**A**^	**66.06 (51.38–85.88)** ^**B**^	0.36	**0.02**	**0.02**

Normally distributed data expressed as mean ± SE

Non-parametric data expressed as median (min-max).

Superscript capital letters (A, B) denote significant differences between groups with different letters where a p-value <0.05 is considered significant

No superscript letters in a row indicates no significant differences between groups for the measured parameter

## Discussion

Metabolomic profiling of obese cats before and after 10 weeks of energy restriction could provide insight on changes in biochemical pathways during energy restriction and allow for more precise and individualized weight loss plan approaches. As previously reported, the metabolomic signature of obesity and energy restriction in strict carnivorous cats differs in certain aspects from omnivorous humans, specifically regarding AA [28]. Whereas BCAA and their catabolites were greater in obese humans undergoing calorie restriction [4], the present study is in agreement with Palloto et al., such that BCAA were not different after weight loss in obese cats [28]. Further, greater BCAA concentrations in humans are potential markers for obesity (8); however, O-MAINT cats in this study had similar serum BCAA concentrations as L-MAINT, suggesting that BCAA concentrations are not good markers for feline obesity. Similar to calorie-restricted cats from Palloto et al., total serum AA concentrations and, specifically, glucogenic AA (alanine, asparagine glutamine, and glycine) were greater in O-RESTRICT compared to O-MAINT and can be indicative of AA oxidation [28]. An increase in AA degradation products and a decrease in citrate also suggests that cats in the O-RESTRICT group had greater AA oxidation to support gluconeogenesis [28].

Cats, as obligate carnivores, have a higher dietary requirement for protein compared to omnivores [43] as a result of routing AA into gluconeogenesis to supply the needs of the brain and other glucose-requiring tissues [44] as well as endogenous nitrogen losses [45]. Cats have shown an ability to adapt AA oxidation rate in response to intake when AA are consumed in sufficient amounts, but may only have a limited ability to adjust AA oxidation when there is a low intake of dietary protein [44, 46]. This could become a disadvantage for cats during periods of starvation or energy restriction as a high rate of AA catabolism could put cats at risk for protein and AA deficiency [47]. Cats in the present study were fed the same diet for the maintenance and restriction periods, thus their protein and AA intake was less during energy restriction. Though previously published by the authors’, protein and individual AA intakes, except for arginine, during energy restriction in the present study, remained above the NRC recommended allowance for adult maintenance [48]. Nonetheless, our knowledge of obligate carnivore AA requirements is limited in contrast to other species. O-RESTRICT cats saw increases in non-essential amino acids, whereas essential AA had no change. The reduction in energy, protein, or individual AA intake could have also affected the partitioning of essential AA, prioritizing essential AA for body processes. Future studies should aim to evaluate changes in the metabolome when protein and AA intake is maintained during restriction.

Further, greater concentrations of serum creatinine, a waste product produced from muscle creatine, was observed in the O-RESTRICT compared to O-MAINT, suggestive of protein catabolism, though it cannot be confirmed for increasing AA oxidation. In the present study, average weight loss rate was 0.94% of initial BW and BCS improved after 10-weeks of restriction; however, use of a more sensitive quantitative method to assess body composition, for example dual-energy x-ray absorptiometry (DXA), would have allowed for more precise assessment of body fat and lean body mass compared to BCS and MCS. Moderate to high protein diets (37.4% DM– 58% DM) during energy restriction are promoted as a way to preserve lean mass through multiple mechanisms such as increasing energy expenditure through a higher thermic effect of feeding [49–51]. However, use of a moderate protein diet (35.9% DM) in overweight and obese cats undergoing energy restriction resulted in loss of lean soft tissue mass, confirmed using DXA, with similar AA metabolism markers; though lean body mass loss was not significant until 16 weeks of restriction suggesting that compared to fat mass loss, lean soft tissue mass loss was slower [11]. Four months of calorie restriction to achieve a weight loss rate of 1% of initial BW in cats consuming a moderate protein diet (38.3% DM) resulted in weight loss from fat mass but also 13% of weight loss was attributed to lean body mass [52]. However, after calorie restriction, cats consuming the moderate protein diet at maintenance for 3 months regained the lean body mass without significant changes in BW. An increase in serum creatinine in the present study with a similar, moderate protein diet (38.6% DM) to the two previous studies during calorie restriction suggests that there could have been a similar loss of lean body mass, though this cannot be confirmed. Additionally, based on the findings from Floerchinger et al., [52] a weight maintenance period following calorie restriction may render the loss of lean body mass insignificant [52], though this was not investigated in the present study.

In adult obese humans and overweight/obese dogs, targeted metabolomic technologies revealed that PC aa C32:1, PC aa C32:2 and PC aa C38:3 were positively associated with overweight or obesity in both humans and dogs, though additional PC were also reportedly elevated in overweight and obese dogs compared to normal weight dogs [18, 53–55]. In humans, PC ae C34:3, PC ae C38:4, and PC ae C40:6 were negatively associated with obesity [53, 56, 57]; however no PC ae were lower in overweight or obese dogs [18]. In the present study, PC ae were similar for O-MAINT and L-MAINT and only two PC aa were significantly elevated in the O-MAINT group: PC aa C32:1, and C38:5. In human obesity, circulating fatty acids are indicative of dyslipidemia. Regarding obese cats in the present study, elevated serum cholesterol was not observed; however, triglycerides were elevated in O-MAINT. Energy restriction also reduced serum triglyceride concentrations in overweight and obese cats previously (25) though this was not observed in the present study. Lysophosphatidylcholines have also been reported to be higher with obesity in some human studies and lower in others [53, 54, 56, 57], though a recent review found that most LPC are negatively associated with body mass index in humans [55]. In overweight/obese dogs, LPC C20:4 was elevated, whereas LPC C14:0, LPC C16:0, and LPC C18:2 were lower [18]. Similarly, to both humans and dogs, LPC C20:4 was elevated in the obese cats in the present study, suggesting that this may also be a marker for feline obesity; however, only one other LPC was elevated, and no other LPC concentrations were different between the obese and lean cats. Multiple PC aa, PC ae, and LPC had lower serum concentrations when obese cats were under calorie restriction in the present study; consistent with previous findings in cats undergoing weight loss via energy restriction [28].

The reduction in various PC in the O-RESTRICT cats could indicate increased transport of fatty acids to the liver to be oxidized for energy [58]. When hydrolyzed to release fatty acids, acylcarnitines are formed. Acylcarnitines are then transported across the mitochondrial membrane via the carnitine-acylcarnitine transporter to be metabolized via beta oxidation. An increase in the concentration of circulating even-chain acylcarnitines (C6 to C22) has previously been associated with incomplete beta oxidation [59, 60]. However, during calorie restriction, an increase in acylcarnitines may indicate an increase in fatty acid oxidation rate rather than incomplete beta oxidation [5]. An increase in even-chain acylcarnitines in O-RESTRICT cats was observed in the present study. Interestingly, O-MAINT did not demonstrate this phenomenon, suggesting that an increase in even-chain acylacarnitines occurred due to energy restriction. Incomplete beta oxidation is unlikely since O-RESTRICT cats had successful weight loss and reductions in BCS, suggesting that body fat mass was reduced. The role of acylcarnitines, specifically during calorie restriction, has not been thoroughly investigated in the cat, an obligate carnivore, compared to humans, and as such, definitive conclusions cannot be made, and further research is needed.

Increased mobilization of fatty acids to the liver from lipolysis during weight loss has previously been reported in cats undergoing weight loss resulting in hepatic lipidosis [48]. The accumulation of long-chain polyunsaturated fatty acids (LCPUFA) in hepatic tissue can contribute to the development of hepatic lipidosis in cats, humans, equine, avian, and reptilian species [48, 61–65]. Obesity is a risk factor for hepatic lipidosis across various species [61, 62, 64, 65], though rapid weight loss with reduced energy intake, or anorexia, is considered the most common cause in cats [59–62]. Increases in some fatty acids, and decreases in others, have been observed in cats with hepatic steatosis after rapid weight loss [66]. Using metabolomic analyses, Palloto et al., also observed a decrease in LCPUFA in calorie restricted cats after 8 weeks of slow weight loss, though cats in both the present study and Palloto’s study did not develop hepatic lipidosis [28]. Additionally, several sphingomyelin metabolites increased in O-RESTRICT compared to O-MAINT which has previously been associated with hepatic lipidosis in cats and animal models of obesity [67–69]. In humans with non-alcoholic fatty liver disease, increased synthesis of sphingomyelin has shown to promote hepatocyte apoptosis as well as release of inflammatory mediators [70]. Since cats in both studies underwent gradual weight loss, and fatty acid mobilization and oxidation appeared to be occurring, it is unlikely that lipid accumulation was sufficient to develop clinical signs for hepatic lipidosis [58, 71]. However, the increase in various metabolites associated with human obesity, insulin resistance, and other metabolic diseases and the potential deficiency in LCPUFA warrants further research to understand the role of PC, LPC, acylcarnitines, and sphingomyelins in obesity, weight loss, and in the pathogenesis of hepatic lipidosis in cats [67].

The observed changes in PC, LPC, sphingomyelin, and acylcarnitines in combination with a reduction of serum insulin, BW, and BCS, suggests that lipolysis is occurring in the O-RESTRICT cats, though there is a lack of evidence to support that lipolysis and ketosis were increased under calorie restriction compared to O-MAINT in the present study. Metabolites involved in these pathways including glycerol, NEFA, L-carnitine, BHB, and acetoacetate were not different between O-MAINT and O-RESTRICT. Previously, Hoenig et al. found that compared to humans, obese cats have greater lipolysis as noted by higher NEFA concentrations as well as clearance of fatty acids compared to lean cats and that lipid oxidation predominated in obese cats compared to glucose oxidation in lean cats [72]; however, this was between lean and obese cats and not during energy restriction. In the present study, serum NEFA concentrations did not differ between groups, though the O-MAINT cats had greater glycerol concentrations than L-MAINT. Glycerol is produced and bound to fatty acids to be stored as adipose tissue; however, it is also a break-down product of lipolysis [4]. In human obesity, glycerol production increases to facilitate adipose tissue production [73], and this may explain the observed increase in O-MAINT cats compared to L-MAINT. Interestingly, while O-RESTRICT cats did not have lower glycerol levels compared to O-MAINT, the calorie-restricted cats had a numerical reduction in serum glycerol concentrations such that they were also similar to the L-MAINT cats. When evaluated in combination with other findings, it is likely that glycerol production for adipose tissue synthesis was reduced, and lipolysis was still occurring under calorie restriction. Further, Palloto et al., observed increases in glycerol suggesting greater lipolysis in overweight and obese cats undergoing weight loss [28]. However, not all cats were considered obese and there was no lean control group, therefore, it is likely that the overweight cats in that study did not have the same increased glycerol production as obese cats in the present study. As such, an increase in glycerol from lipolysis could be identified in that study. Additionally, as other markers of lipolysis were not different between O-MAINT and O-RESTRICT, these findings suggest that the cats were mobilizing lipid to meet the needs of calorie restriction via lipolysis.

The ketone body metabolites BHB, acetoacetate, and acetone are produced in a state of negative energy balance [74]. Though acetoacetate or acetone did not differ, BHB was greater in O-MAINT and O-RESTRICT compared to L-MAINT. Changes in BHB in response to macronutrient composition, without changes in energy intake, have previously been reported in cats [7]. In the present study, O-MAINT and L-MAINT cats were consuming the same diet to maintain BW. These findings could be due to the response of BHB to differences in total energy, and macronutrient, intake. Additionally, increased BHB has been suggested as a potential biomarker for obesity and obesity-associated disorders such as hepatic lipidosis and insulin resistance in cats [67, 74, 75]. The O-MAINT cats in the present study did not present with clinical signs for hepatic lipidosis or insulin resistance; however, increased serum BHB and insulin was observed which could indicate greater propensity for insulin resistance in the O-MAINT cats, though this cannot be confirmed. Previously, in calorie-restricted overweight and obese cats, BHB and acetoacetate were elevated, which, in combination with lowered circulating insulin and triglycerides, suggests a shift to fatty acid oxidation, utilizing lipids and ketones for energy [28]. In the present study, O-RESTRICT cats lost BW and, as previously discussed, lipolysis was likely occurring, suggesting that these findings are in line with Pallotto et al. [28].

The comparison between L-MAINT and O-RESTRICT needs to be interpreted with caution as O-RESTRICT cats had restricted energy intake and were on a weight loss plan for 10 weeks, though no cat reached its ideal BW or an ideal BCS during this time. Additionally, O-RESTRICT cats had reduced overall nutrient intake compared to L-MAINT and O-MAINT. Therefore, this comparison is difficult to interpret. A longer-term study in which cats reach their ideal BW may provide deeper insights. Conducting this study with client owned cats also comes with limitations that are related to the cat owners and cannot be controlled. For example, the length of fasting before sampling could have been altered by the cat owners. Cat owners were also not given instructions with respect to meal frequency, which has previously been shown to have a role in plasma AA concentrations in cats [76]. In future studies that investigate the effects of energy restriction on serum metabolome, meal frequency and length of overnight fasting should be consistent. Additionally, owner compliance is a concern for client-owned research and within veterinary practice [77–80]. In the present study, owners maintained and recorded food intake in accordance with the requirements for the study; however, owners may not have recorded all non-compliance measures such as treat administration, stealing food items, and access to other pets’ food in the household. Furthermore, this study assessed the metabolome of calorie restriction using one veterinary extruded dry food. While dry food is the most common diet purchased by pet owners, it is common for cat owners to also feed wet food [77–79] and it is a common recommendation for obese cats undergoing weight loss to consume a food higher in moisture [36]. Therefore, additional studies investigating the metabolomic response to calorie restriction in cats should be conducted comparing weight loss across multiple dietary formats, such as dry, wet, or alternative pet foods.

## Conclusions

The current study provides evidence to support the fact that energy restriction in obese cats using a veterinary food formulated for maintenance and weight loss results in increased protein catabolism and AA partitioning to meet energy requirements and appeared to induce changes in fatty acid oxidation rate. Future studies should aim to elucidate the role of acylcarnitine, LPC, sphingomyelins, and ketone bodies in obese cats and cats under calorie restriction as well as to evaluate the effect of various levels of dietary protein and amino acid intake, proportions of weight loss from fat tissue and lean body tissue during weight loss, and in combination with lipotropic supplementation such as L-carnitine, choline, or the addition of LCPUFA.
